# Cortical Excitability and Activation of TrkB Signaling During Rebound Slow Oscillations Are Critical for Rapid Antidepressant Responses

**DOI:** 10.1007/s12035-018-1364-6

**Published:** 2018-10-04

**Authors:** Samuel Kohtala, Wiebke Theilmann, Marko Rosenholm, Leena Penna, Gulsum Karabulut, Salla Uusitalo, Kaija Järventausta, Arvi Yli-Hankala, Ipek Yalcin, Nobuaki Matsui, Henna-Kaisa Wigren, Tomi Rantamäki

**Affiliations:** 1 Faculty of Biological and Environmental Sciences, University of Helsinki, P.O.Box 65, Viikinkaari 1, Helsinki, Finland; 2 Laboratory of Neurotherapeutics, Faculty of Pharmacy, Division of Pharmacology and Pharmacotherapy, University of Helsinki, Viikinkaari 5, P.O. Box 56, Helsinki, Finland; 3 Institut des Neurosciences Cellulaires et Intégratives, Centre National de la Recherche Scientifique and Université de Strasbourg, Strasbourg Cedex, France; 4 Department of Anesthesiology and Reanimation, Gazi University, Ankara, Turkey; 5 Department of Psychiatry, Tampere University Hospital, Tampere, Finland; 6 Faculty of Medicine and Life Sciences, Department of Anesthesiology, University of Tampere, Tampere, Finland; 7 Department of Anaesthesia, Tampere University Hospital, Tampere, Finland; 8 Faculty of Pharmaceutical Sciences, Tokushima Bunri University, Tokushima, Japan; 9 Faculty of Medicine, Medicum/Physiology, University of Helsinki, Helsinki, Finland

**Keywords:** Rapid-acting antidepressant, Nitrous oxide, Ketamine, Cortical excitation, Electroencephalogram, Sedation

## Abstract

Rapid antidepressant effects of ketamine become most evident when its psychotomimetic effects subside, but the neurobiological basis of this “lag” remains unclear. Laughing gas (N_2_O), another NMDA-R (*N*-methyl-d-aspartate receptor) blocker, has been reported to bring antidepressant effects rapidly upon drug discontinuation. We took advantage of the exceptional pharmacokinetic properties of N_2_O to investigate EEG (electroencephalogram) alterations and molecular determinants of antidepressant actions during and immediately after NMDA-R blockade. Effects of the drugs on brain activity were investigated in C57BL/6 mice using quantitative EEG recordings. Western blot and qPCR were used for molecular analyses. Learned helplessness (LH) was used to assess antidepressant-like behavior. Immediate-early genes (e.g., *bdnf*) and phosphorylation of mitogen-activated protein kinase—markers of neuronal excitability—were upregulated during N_2_O exposure. Notably, phosphorylation of BDNF receptor TrkB and GSK3β (glycogen synthase kinase 3β) became regulated only gradually upon N_2_O discontinuation, during a brain state dominated by slow EEG activity. Subanesthetic ketamine and flurothyl-induced convulsions (reminiscent of electroconvulsive therapy) also evoked slow oscillations when their acute pharmacological effects subsided. The correlation between ongoing slow EEG oscillations and TrkB-GSK3β signaling was further strengthened utilizing medetomidine, a hypnotic-sedative agent that facilitates slow oscillations directly through the activation of α_2_-adrenergic autoreceptors. Medetomidine did not, however, facilitate markers of neuronal excitability or produce antidepressant-like behavioral changes in LH. Our results support a hypothesis that transient cortical excitability and the subsequent regulation of TrkB and GSK3β signaling during homeostatic emergence of slow oscillations are critical components for rapid antidepressant responses.

**Electronic supplementary material:**

The online version of this article (10.1007/s12035-018-1364-6) contains supplementary material, which is available to authorized users.

## Introduction

Major depression is a highly disabling condition, the most significant risk factor for suicide and one of the biggest contributors to the global disease burden [1]. Many patients respond poorly to standard antidepressants, and with those who do respond, the therapeutic effects become evident with a considerable delay. Furthermore, the clinical diagnosis of major depression and the treatments in current use are lacking objective biomarkers [2].

The remarkable ability of NMDA-R (*N*-methyl-d-aspartate receptor) blocker ketamine, a dissociative anesthetic drug, to ameliorate depressive symptoms rapidly after a single subanesthetic intravenous infusion has stimulated great enthusiasm among scientists and clinicians [3, 4]. Reported response rates to ketamine are impressive, but many patients remain treatment-refractory [3]. Therefore, extensive research efforts have been invested to find predictive efficacy markers and to uncover the precise pharmacological basis governing ketamine’s antidepressant effects. Experimental evidence suggests that ketamine increases glutamate release and enhances glutamatergic AMPA-R (α-amino-3-hydroxy-5-methyl-4-isoxazolepropionic acid receptor) function, which in turn augments synaptic plasticity through the BDNF (brain-derived neurotrophic factor) receptor TrkB [5–10]. Indeed, positive allosteric AMPA-R modulators increase BDNF synthesis in the brain and produce antidepressant-like effects in rodents [11]. Inhibition of GSK3β (glycogen synthase kinase 3β), another molecular event tightly connected with ketamine’s therapeutic effects [12], also contributes to the enhanced AMPA-R function [13].

The antidepressant effects of subanesthetic ketamine become most evident when its psychotropic actions and acute pharmacological effects on NMDA-R fade [3, 14], but the neurobiological basis of this “lag” remains unclear. Indeed, after systemic administration, ketamine is readily distributed in the body and it undergoes rapid elimination and metabolism [15]. Notably, a recent animal study suggests that some of the metabolites of ketamine, especially hydroxynorketamines that preferentially act through AMPA-R, may account for the antidepressant effects of ketamine [16] (However, see 17). This hypothesis, however, conflicts with earlier investigations emphasizing the critical role of NMDA-R blockade and the promising clinical observations with some other NMDA-R antagonists in depressed patients [18]. Of these agents, nitrous oxide [19] (N_2_O, “laughing gas”) is particularly interesting since (in both mice and humans) it has extremely fast kinetics and is essentially not metabolized in the body. Notably, in this small pilot study conducted by Nagele and colleagues [20], the antidepressant effects of N_2_O were observed few hours after the gas administration, a time period when the drug has been essentially eliminated from the body. Moreover, rapid improvement of depression has been occasionally reported after electroconvulsive therapy (ECT), a non-pharmacological treatment where an electric pulse is delivered into the scalp to induce transient epileptiform activity in the EEG (electroencephalogram) [21]. Analogous effects are produced by some pharmacological convulsants such as GABA_A_-R (γ-aminobutyric acid receptor type A) antagonist flurothyl [22–24], which are however no longer used in clinical practice.

Postictal (i.e., after seizure) emergence of slow EEG activity within the delta (1–4 Hz) and theta range (4–7 Hz) and/or burst suppression pattern has been associated with the efficacy and onset-of-action of “convulsive therapies” [25]. Interestingly, acute administration of subanesthetic doses of NMDA-R antagonists ketamine and MK-801 has also been associated with gradual increases in slow EEG activity [26–28]. These slow oscillations are thought to emerge as a homeostatic response of neuronal networks to the preceding cortical excitation, which in the case of ketamine is suggested to result from the preferential inhibition of NMDA-R located on inhibitory interneurons and the following disinhibition of pyramidal neurons [9, 29, 30]. Notably, slow oscillations are characteristic of deep NREM (non rapid-eye movement) sleep and can also be increased directly and without preceding cortical excitation with diverse hypnotic/sedative agents [31]. One of these drugs is medetomidine, a selective α_2_-adrenergic autoreceptor agonist commonly used to produce sedation and anesthesia in veterinary medicine.

To provide a better understanding of the rapid antidepressant mechanisms, we took advantage of the rapid pharmacokinetic and dynamic properties of N_2_O to investigate potential shared EEG alterations and the regulation of the molecular determinants of antidepressant actions during and after NMDA-R blockade. Our findings suggest that N_2_O, similarly to that with subanesthetic ketamine and fluorothyl, produces a transient period of cortical excitation during gas administration which is followed by rebound emergence of homeostatic slow EEG oscillations after the gas flow is suspended. Most interestingly, TrkB and GSK3β signaling alterations remain unchanged during N_2_O exposure but are evoked gradually upon gas discontinuation along with slow oscillations. The positive correlation between the emergence of slow EEG oscillations and TrkB and GSK3β signaling was further strengthened with medetomidine. Medetomidine did not, however, facilitate markers of neuronal excitability or produce antidepressant-like behavioral changes in LH. This study supports a hypothesis that transient cortical excitability and the subsequent regulation of TrkB and GSK3β signaling during rebound slow oscillations are critical components for rapid antidepressant responses.

## Methods and Materials

### Animals

Adult C57BL/6JRccHsd mice (Harlan Laboratories, Venray, the Netherlands) were used. Animals were maintained in the animal facility of University of Helsinki, Finland, under standard conditions (21 °C, 12-h light-dark cycle) with free access to food and water. The experiments were carried out according to the guidelines of the Society for Neuroscience and were approved by the County Administrative Board of Southern Finland (License: ESAVI/10527/04.10.07/2014).

### Pharmacological Treatments and Sample Collection

Medical grade N_2_O (Livopan 50% N_2_O/O_2_ mix, Linde Healthcare; Niontix 100% N_2_O, Linde Healthcare) and medical grade oxygen (Conoxia 100% O_2_, Linde Healthcare) were mixed with 100% N_2_O to achieve > 50% N_2_O concentrations. After habituation to the experimental conditions, the gas was administered into airtight Plexiglass chambers (for biochemical analyses (width × length × height): 14 cm × 25 cm × 9 cm; for biochemical and EEG analyses: 11.5 cm × 11.5 cm × 6.5 cm) with a flow rate of 4–8 l/min. O_2_ or room air was used as control gas.

To induce myoclonic seizures, 10% flurothyl liquid (in 90% ethanol; Sigma-Aldrich) was administered into the cotton pad placed inside the lid of an airtight Plexiglass chamber (13 cm × 13 cm × 13 cm) at the flow rate of 100–200 μl/min until the mice exhibited seizures. The lid was removed to terminate the seizure. Animals were euthanized at indicated times post-seizure. Ethanol solution was given for the sham animals.

Ketamine-HCl (7.5–10 mg/kg; Ketaminol®, Intervet International B.V.) and medetomidine-HCl (0.05–0.3 mg/kg, i.p./s.c.; Domitor®, Orion Pharma) were diluted in isotonic saline solution and injected intraperitoneally with an injection volume of 10 ml/kg.

Animals were euthanized at indicated times after the treatments by rapid cervical dislocation followed by decapitation. No anesthesia was used due to its potential confounding effects on the analyses [32, 33]. Bilateral medial prefrontal cortex (including prelimbic and infralimbic cortices) was rapidly dissected on a cooled dish and stored at − 80 °C.

### Western Blotting and Quantitative RT-PCR

For western blotting, the brain samples were homogenized in lysis buffer (137 mM NaCl, 20 mM Tris, 1% NP-40, 10% glycerol, 48 mM NaF, H_2_O, Complete inhibitor mix (Roche), PhosStop (Roche)) [33]. After ~ 15 min incubation on ice, samples were centrifuged (16,000×*g*, 15 min, + 4 °C) and the resulting supernatant collected for further analysis. Sample protein concentrations were measured using Bio-Rad DC protein assay (Bio-Rad Laboratories, Hercules, CA). Proteins (40–50 μg) were separated with SDS-PAGE under reducing and denaturing conditions and blotted to a PVDF membrane as described. Membranes were incubated with the following primary antibodies (see (32)): anti-p-TrkB (#4168; 1:1000; Cell signaling technology (CST)), anti-TrkB (1:1000; #4603, CST), anti-Trk (sc-11; 1:1000; Santa Cruz Biotechnology (SCB);), anti-p-CREB (#9191S; 1:1000; CST), anti-p-p70S6K (#9204S; 1:1000; CST), anti-p-GSK3βS9 (#9336; 1:1000; CST), anti- p-p44/42-MAPKThr202/Y204 (#9106, 1:1000, CST), anti-GSK3β (#9315, 1:1000, CST), anti-p70S6K (#2708, 1:1000, CST) anti-p44/42-MAPK (#9102, 1:1000, CST), and anti-GAPDH (#2118, 1:10000, CST). Further, the membranes were washed with TBS/0.1% Tween (TBST) and incubated with horseradish peroxidase conjugated secondary antibodies (1:10000 in non-fat dry milk, 1 h at room temperature; Bio-Rad). After subsequent washes, secondary antibodies were visualized using enhanced chemiluminescence (ECL Plus, ThermoScientific, Vantaa, Finland) for detection by Biorad ChemiDoc MP camera (Bio-Rad Laboratories, Helsinki, Finland).

For qPCR, total RNA of the sample was extracted using Trizol (Thermo Scientific) according to the manufacturer’s instructions and treated with DNAse I mix. mRNA was reverse transcribed using oligo (dT) primer and SuperScript III Reverse Transcriptase mix (Thermo Scientific). The amount of cDNA was quantified using real-time PCR. The primers used to amplify specific cDNA regions of the transcripts are shown in Table S1. DNA amplification reactions were run in triplicate in the presence of Maxima SYBRGreen qPCR mix (Thermo Scientific). Second derivate values from each sample were obtained using the LightCycler 480 software (Roche). Relative quantification of template was performed as described previously using standard curve method, with cDNA data being normalized to the control *Gapdh* and *ß-actin* level.

### EEG Recordings and Data Analysis

For the implantation of electrodes, mice were anesthetized with isoflurane (3% induction, 1.5–2% maintenance). Lidocaine (10 mg/ml) was used as local anesthetic and buprenorphine (0.1 mg/kg, s.c.) for postoperative care. Two epidural screw EEG (electroencephalogram) electrodes were placed above the fronto-parietal cortex. A further screw served as mounting support. Two silver wire electrodes were implanted in the nuchal muscles to monitor the EMG (electromyogram). After the surgery, mice were single-housed in Plexiglas boxes. After a recovery period of 5–7 days, animals were connected to flexible counterbalanced cables for EEG/EMG recording and habituated to recording cables for 3 days.

Baseline EEG (10–15 min) recordings of awake animals were conducted prior the treatments. All injection treatments were conducted in the animal’s home cages during light period. N_2_O treatment was delivered in homemade anesthesia boxes for indicated time periods with a flow rate of 8 l/min. The EEG and EMG signals were amplified (gain 5 or 10 K) and filtered (high pass: 0.3 Hz; low pass 100 Hz; notch filter) with a 16-channel AC amplifier (A-M System, model 3500), sampled at 254 Hz or 70 Hz with 1401 unit (CED), and recorded using Spike2 (version 8.07, Cambridge Electronic Devices). The processing of the EEG data was obtained using Spike2 (version 8.07, Cambridge Electronic Devices). EEG power spectra were calculated within the 1–50 Hz frequency range by fast Fourier transform (FFT = 256, Hanning window, 1.0 Hz resolution). Oscillation power in each bandwidth (delta = 1–4 Hz; theta = 4–7 Hz; alpha = 7–12 Hz; beta = 12–25 Hz; gamma low = 25–40 Hz; gamma high = 60–100 Hz) was computed in 30–300-s epochs from spectrograms (FFT size: 1024 points) for each animal. Representative sonograms were computed using a Hanning window with a block size of 512.

### Learned Helplessness

Animals were placed in a shuttle box (Panlab LE100-26, LE900; Software: Bioseb Packwin) and let habituate for 3 min. On day 1, a pre-test was conducted consisting of 140 randomly paced (at 25, 30, or 35 s intervals) inescapable foot shocks (0.45 mA, 20 s duration). The pre-test was repeated on day 2. On day 3, testing was conducted starting with 1-min habituation and followed by 15 randomly paced (at 25, 30, or 35 s intervals) escapable shocks (0.45 mA, 20 s duration). During testing, animals were able to interrupt the shock delivery/escape by crossing to another chamber. If the animal failed to escape during the first 10 s of a test shock, the trial was considered as a failure. If more than 50% of the 15 trials led to a failure, the animal was considered helpless. After testing, animals were injected (i.p.) with saline, ketamine (15 mg/kg), or medetomidine (0.05 mg/kg). Learned helplessness was re-evaluated 24 h post-injection.

### Statistical Analyses

Depending on whether data were normally distributed or not, either parametric or nonparametric test was used for statistical evaluation. In case of more than two groups, analysis of variance (ANOVA) with post hoc test was used. All statistical analyses were performed with the Prism 7 software from GraphPad (La Jolla, CA, USA). All tests were two-sided; a *P* ≤ 0.05 was considered significant. Details of statistical tests and *n* numbers for each experiment are shown in Table S2.

## Results

### Markers of Neuronal Excitability Are Upregulated During N_2_O Exposure

**Fig. 1:**
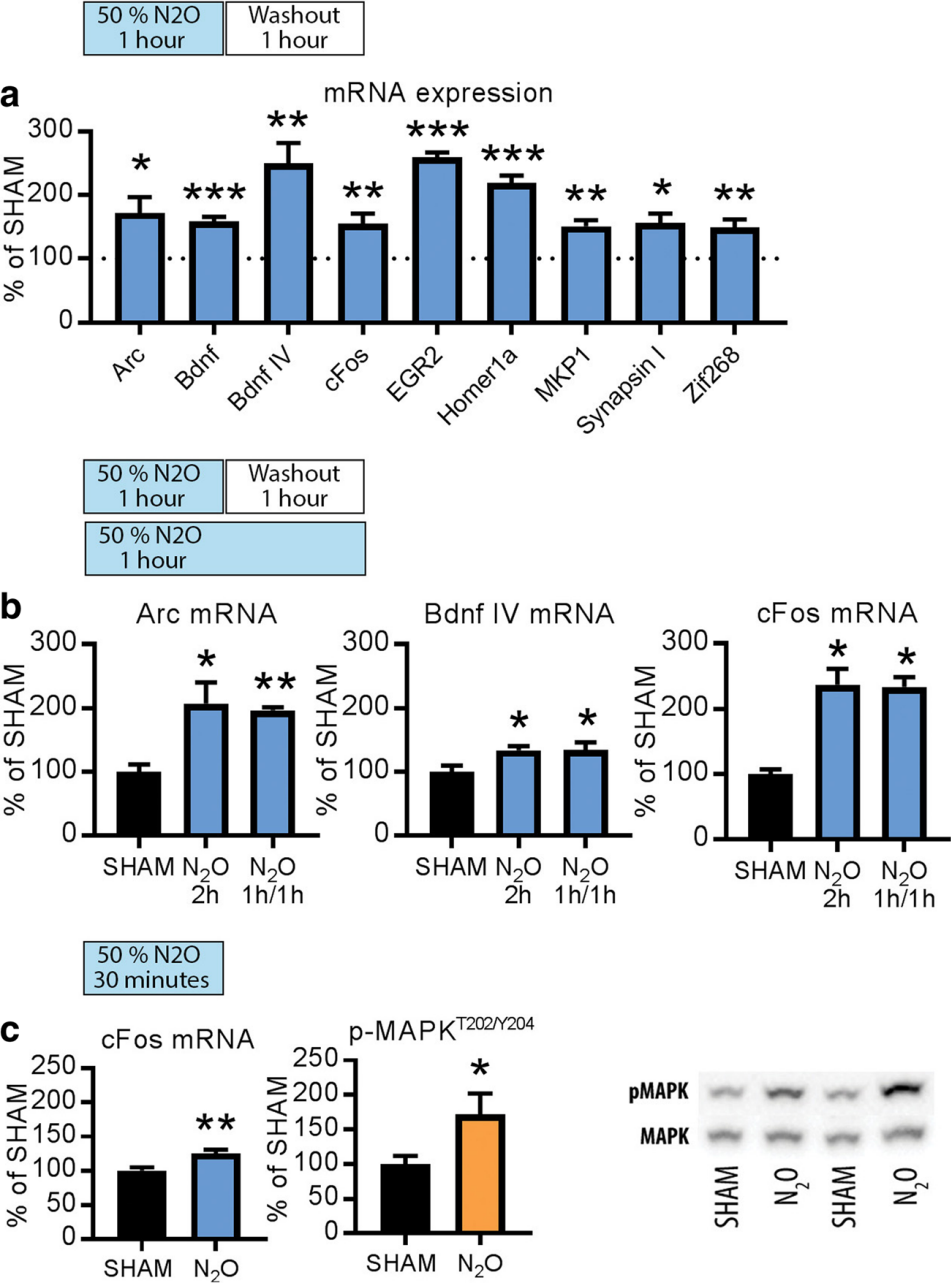
**a** Levels of *arc*, *bdnf*, *c-fos, egr-2, homer-1a, mkp-1, synapsin 1*, and *zif-268* mRNA after continuous administration of N_2_O (50%) for 1 h and a 1-h washout period. **b** *arc, bdnf*, and *c-fos* mRNA levels are similarly upregulated by 2-h continuous N_2_O (50%) and 1-h N_2_O (50%) followed by a 1-h washout period. **c** *c-fos* mRNA and p-MAPK^T202/Y204^ levels are increased after 30 min of N_2_O (50%) administration. Data are means ± S.E.M. *< 0.05, **< 0.01, ***< 0.001 (for statistical analyses and *n* numbers, see Table S2)

Clinically effective rapid-acting antidepressants have the capacity to rapidly yet transiently increase cortical excitability. To test whether N_2_O produces similar effects, we adopted the treatment protocol used in the clinical study by Nagele et al. [20] and investigated how biological markers associated with neuronal excitability are regulated. Mice received continuous 50% of N_2_O for an hour after which the animals breathed room air for another hour. We focused our analyses to the medial prefrontal cortex (mPFC), a brain region associated in the pathophysiology of depression and antidepressant actions. This N_2_O treatment increased the expression of several mRNAs of activity-dependent immediate-early genes (*c-fos*, *arc*, *bdnf*, *zif-268, homer-1A, egr-2, mkp-1, synapsin*) (Fig. 1a).

To investigate whether these responses appear during N_2_O exposure or withdrawal, we carried out an experiment in which a subgroup of animals breathed contiously 50% N_2_O for 2 h and samples collected immediately thereafter. Importantly, *arc, bdnf*, and *c-fos* mRNA levels were readily upregulated also by this treatment (Fig. 1b). Moreover, *c-fos* mRNA levels and phosphorylated mitogen-activated protein kinase (MAPK^T202/Y204^), another marker of increased neuronal excitability, increased already 30 min after the onset of N_2_O administration (Fig. 1c). Altogether, these data indicate the facilitation of cortical activity under N_2_O. Notably, these acute changes induced by N_2_O resemble those produced by electroconvulsive therapy (ECT) [34, 35], and sleep deprivation [36], which also rapidly alleviate depression in a subset of patients.

### Subanesthetic Ketamine and N_2_O Evoke Rebound Slow EEG Activity Upon Drug Discontinuation

**Fig. 2:**
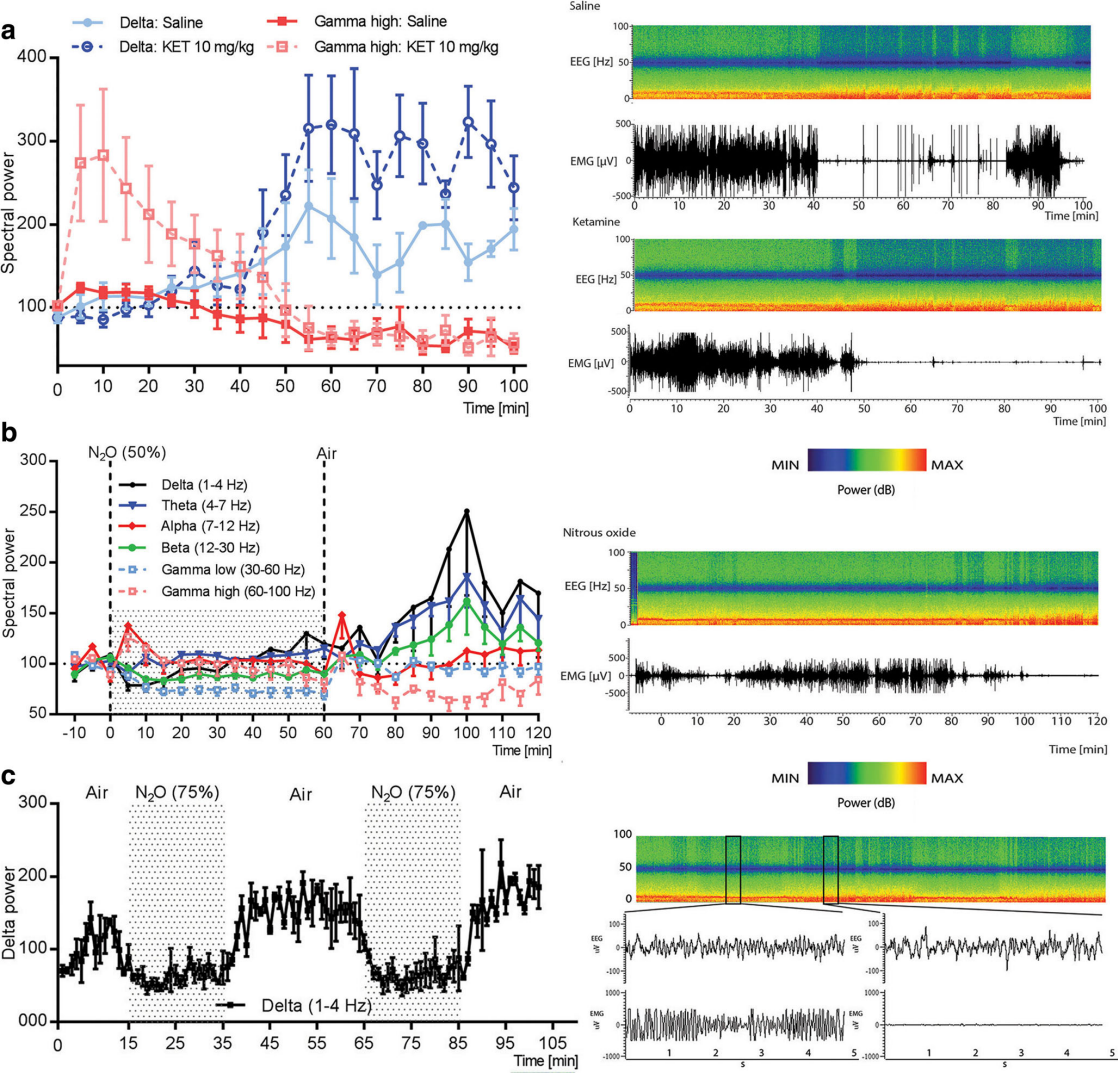
**a** Representative time frequency EEG spectrogram and normalized power of EEG oscillations after a subanesthetic dose of ketamine (KET; 10 mg/kg, i.p.) Subanesthetic ketamine evokes rebound delta oscillations gradually after the acute effects of the drug on high gamma oscillations have dissipated. **b** Slow wave delta (1–4 Hz) and theta (4–7 Hz) EEG oscillations are transiently increased upon N_2_O (50%) withdrawal. **c** Rebound delta oscillations after discontinuation of 75% N_2_O treatment. Data are means ± S.E.M. (for *n* numbers, see Table S2)

Acute administration of low doses of NMDA-R antagonists has been associated with gradual increase in slow EEG activity after the acute effects on cortical excitability subside. Similarly to what has been reported earlier [37], a subanesthetic dose of ketamine initially increased gamma oscillations (Fig. 2a; see also Fig. S1), a neurophysiological sign of ongoing cortical excitability, which lasted around 30–50 min. After this time period, i.e., after the peak of ketamine’s pharmacological effects (serum *t*_1/2_ (mouse): ~ 15 min, see [38], slow-wave delta oscillations gradually increased above baseline compared to saline treated controls. The ability of N_2_O to regulate various biological markers associated with neuronal excitability encouraged us to test whether similar phenomenon might occur following the treatment to N_2_O. Apart from the dampening of low gamma oscillations, no clear EEG alterations were observed during exposure of 50% N_2_O (Fig. 2b). Upon gas withdrawal, however, slow EEG oscillations increased above baseline values. The peak of slow-wave delta emerged at around 40 min post-N_2_O and reduced thereafter towards baseline.

The duration of exposure and concentration of inspired N_2_O can be easily controlled by mixing it with oxygen to varying degrees. We therefore tested if short exposures to higher N_2_O concentrations evoke more substantial increase in slow EEG oscillations following drug discontinuation. Indeed, slow EEG oscillations, especially within the delta range, increased rapidly after a 20-min exposure to 75% N_2_O in both male and female mice and such effect could be rapidly reproduced with intermittent dosing (Fig. 2c; Fig. S1). Beta and low gamma oscillations were reduced during such N_2_O treatments, but these alterations normalized upon gas withdrawal (Fig. S2).

### TrkB and GSK3β Signaling Alterations Emerge During Rebound Slow EEG Activity

**Fig. 3:**
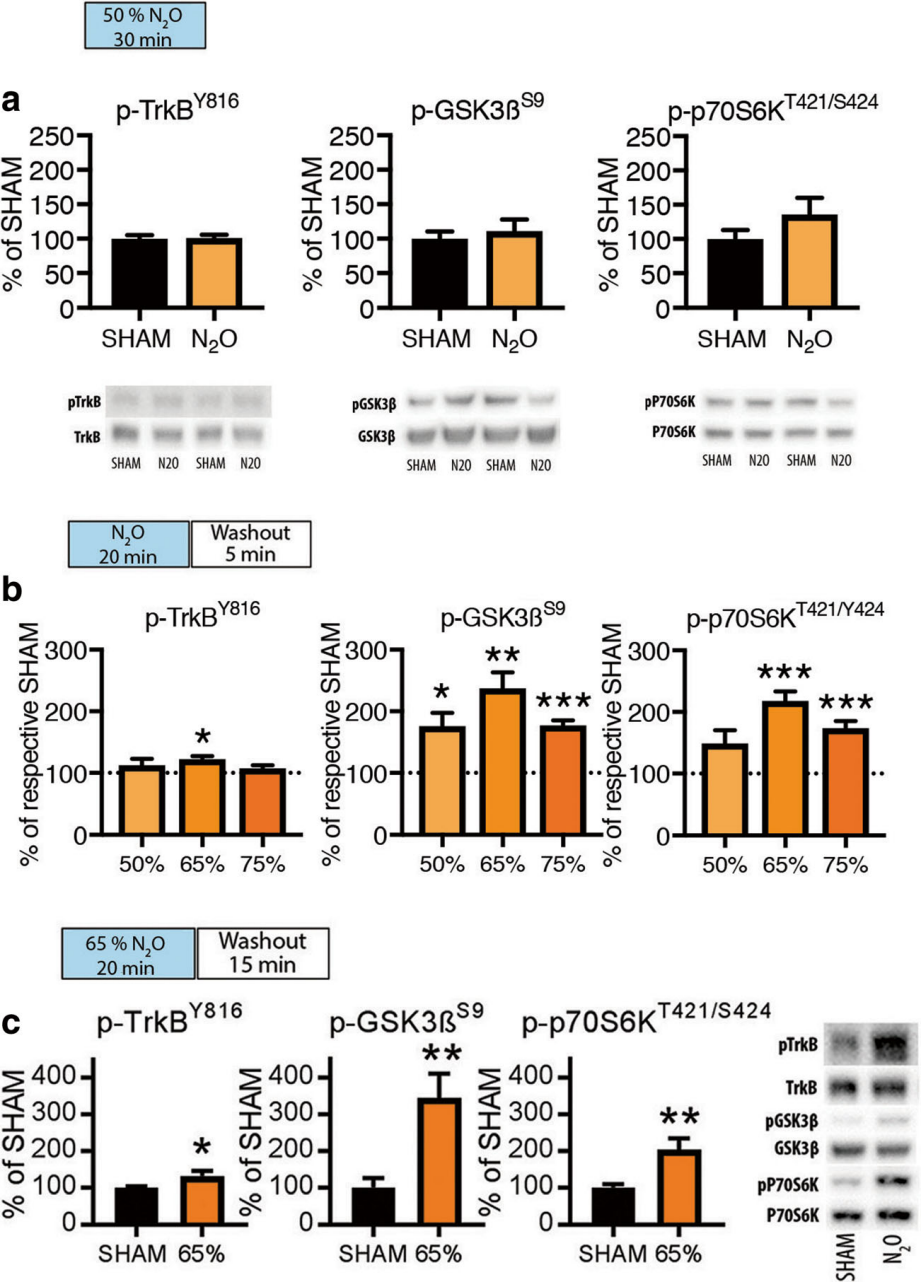
**a** Levels of p-TrkB^Y816^, p-GSK3β^S9^, and p-p70S6k^T421/424^ after 30 min of N_2_O (50%) administration. **b** Levels of p-TrkB^Y816^, p-GSK3β^S9^, and p-p70S6k^T421/424^ at 5-min post-N_2_O exposure (50–75%). **c** Levels of p-TrkB^Y816^, p-GSK3β^S9^, and p-p70S6k^T421/424^ at 15-min post-N_2_O exposure (65%). Data are means ± S.E.M. *< 0.05, **< 0.01, ***< 0.001 (for statistical analyses and *n* numbers, see Table S2)

Activation of BDNF receptor TrkB has been causally connected with antidepressant effects in rodents [39, 40]. Upon activation, TrkB receptors undergo phosphorylation within specific tyrosine residues within the intracellular domain [8, 39, 41–43]. These effects set forth regulation of several intracellular cascades [42] of which activation of MAPK [44] (see Fig. 1) and mTor (mammalian target of rapamycin) [5], and inhibition of GSK3β [12] has been strongly implicated in ketamine’s antidepressant effects. We thus next sought to investigate how TrkB, mTor, and GSK3β phosphorylation are regulated during N_2_O exposure and withdrawal. We first exposed animals to N_2_O for a period of 30-min and collected brain samples immediately thereafter. Interestingly, phosphorylation of TrkB^Y816^, phosphorylation of GSK3β at the inhibitory serine-9 residue (GSK3β^S9^), and phosphorylation of p70S6k^T421/S424^ (downstream target of mTor) remained unaltered in these samples indicating that ongoing NMDA-R blockade is not directly associated with TrkB and GSK3β signaling alterations (Fig. 3a), although MAPK phosphorylation is concomitantly increased.

To test the possibility that TrkB and GSK3β signaling alterations become evident upon withdrawal of N_2_O exposure (i.e., after NMDA-R blockade), we collected brain samples for western blot analyses 5 and 15 min after exposing the animals to varying N_2_O concentrations (50–75%) for 20 min. These data, shown in Fig. 3b, c, indicate that N_2_O can indeed induce TrkB^Y816^ and GSK3β^S9^ phosphorylation but only upon gas withdrawal when slow EEG oscillations become also facilitated.

### TrkB and GSK3β Signaling During Postictal State Induced by Flurothyl

**Fig. 4:**
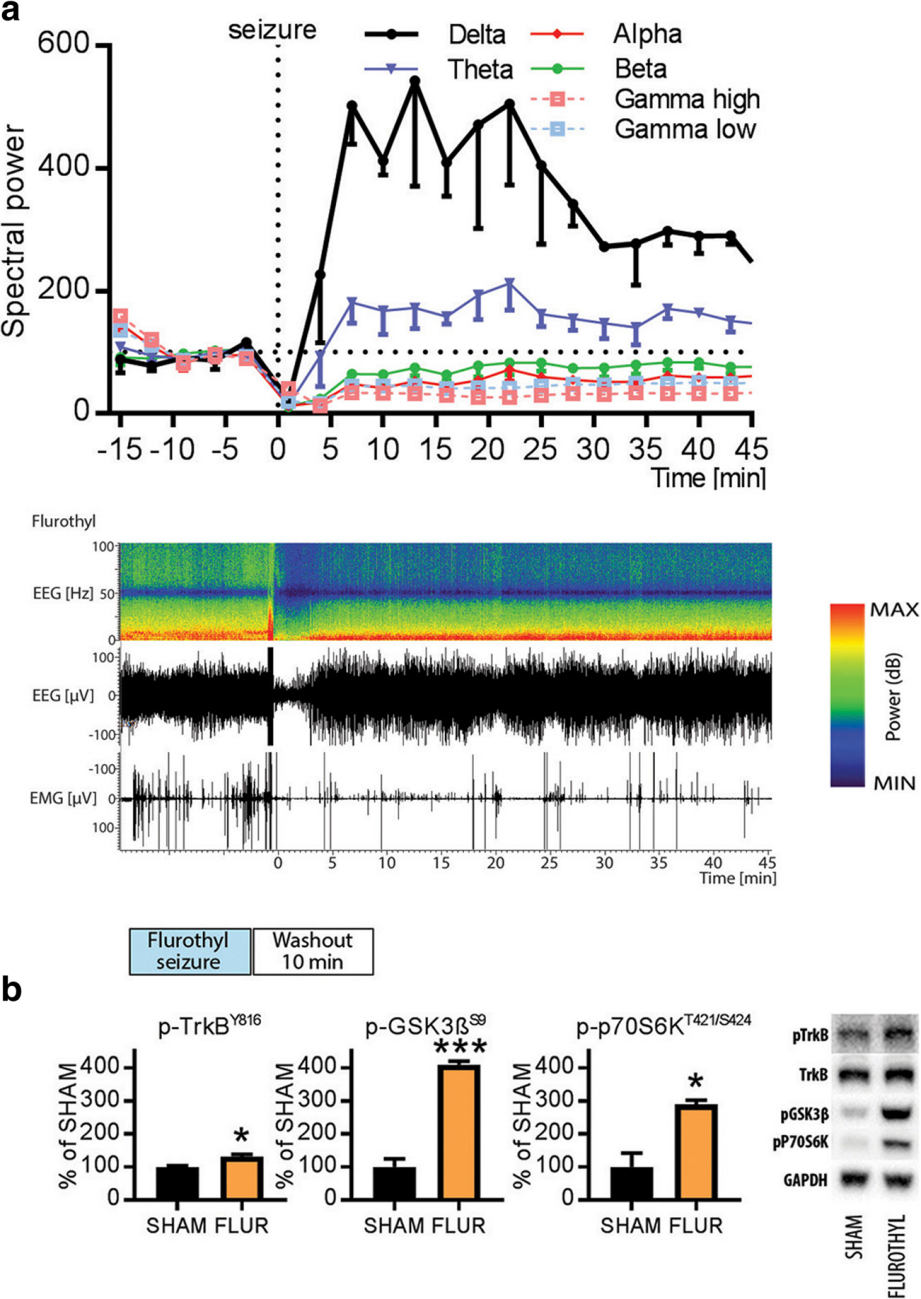
**a** Representative time frequency EEG spectrogram and normalized power of major EEG oscillations after flurothyl-induced seizures. Flurothyl evokes rebound emergence of slow-wave delta and theta oscillations. **b** Levels of p-TrkB^Y816^, p-GSK3β^S9^, and p-p70S6k^T421/424^ 10 min after flurothyl (FLUR) administration. Data are means ± S.E.M. *< 0.05, ***< 0.001 (for statistical analyses and *n* numbers, see Table S2)

Postictal emergence of slow EEG oscillation is also observed in patients after the delivery of flurothyl or ECT [22], and this phenomenon has been considered to predict the efficacy and onset-of-action of convulsive therapies [25]. We sought to recapitulate this in rodents and to further test whether TrkB and GSK3β signaling is altered during a brain state dominated by slow EEG activity. Flurothyl was evaporated into the cage until the mice exhibited a generalized seizure, which terminated within seconds upon drug withdrawal. A robust increase in slow EEG oscillations, particularly within the delta range (1–4 Hz), emerged gradually and peaked within 10 min after the flurothyl-induced seizure (Fig. 4a). Notably, alpha oscillations (7–12 Hz), beta oscillations (12–30 Hz), and high-frequency gamma oscillations (> 25 Hz) were reduced during the postictal period. At the behavioral level, the mice appeared motionless and sedated; a state also correlated with reduced electromyogram (EMG) activity (Fig. 4a). Most importantly, phosphorylation levels of TrkB^Y816^, p70S6k^T421/S424^, and GSK3β^S9^ were robustly elevated in samples collected 10 min after flurothyl (Fig. 4b). Collectively, our data so far suggests that rapid-acting antidepressants evoke TrkB and GSK3β signaling alterations during slow EEG oscillations that are generated as a withdrawal rebound response to the transient increase of cortical excitability induced by the drugs.

### Towards a Homeostatic Basis of Rapid Antidepressant Effects

**Fig. 5:**
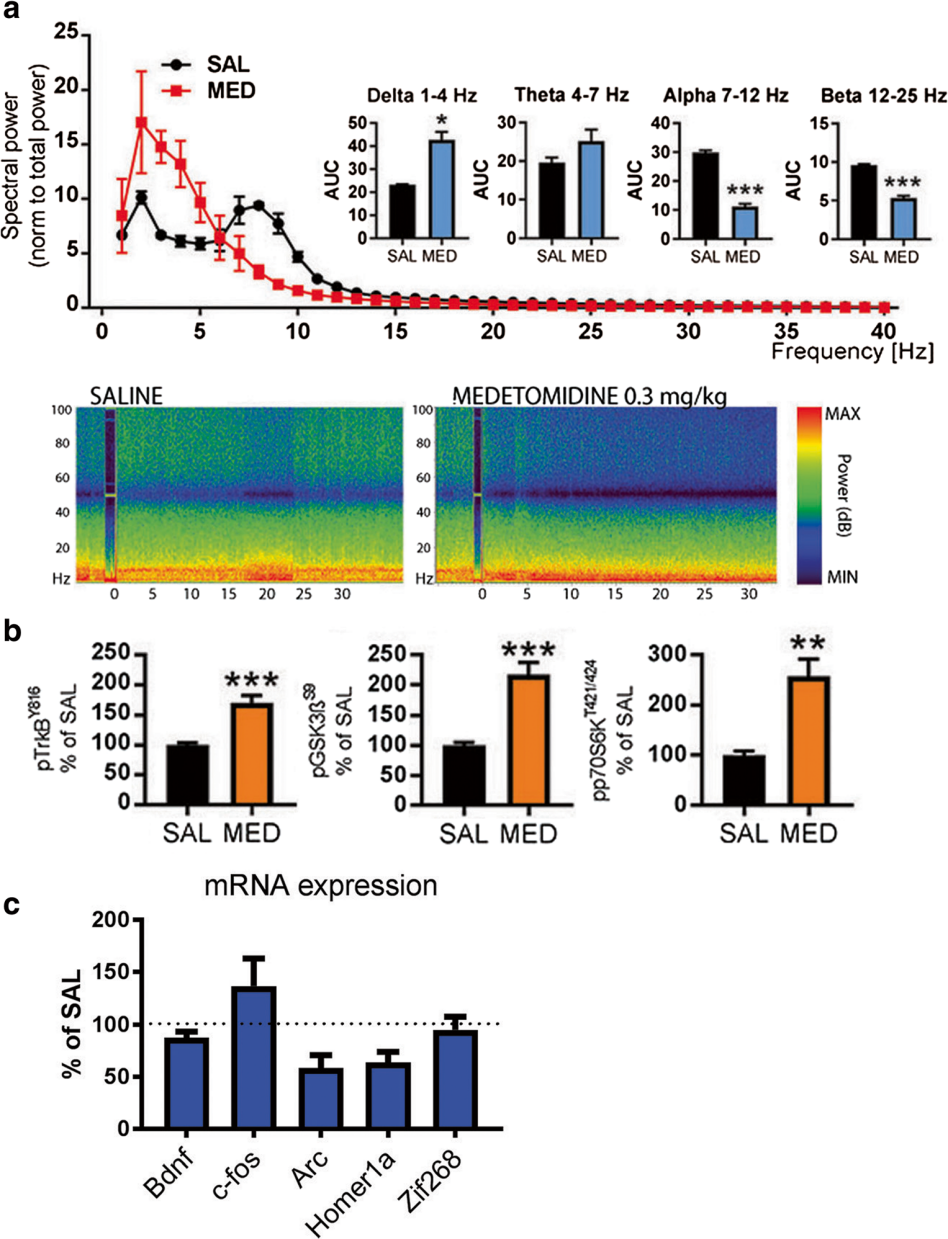
**a** Representative time frequency EEG spectrograms and normalized power of major EEG oscillations during 30-min saline and medetomidine (MED; 0.3 mg/kg, i.p.) treatment. **b** A low dose of medetomidine (0.05 mg/kg, i.p.) rapidly increases phosphorylation of TrkB^Y816^, GSK3β^S9^, and p70S6k^T421/424^ in the mouse medial prefrontal cortex. **c** Levels of *c-fos*, *arc*, *bdnf*, *zif-268, homer-1A, egr-2, mkp-1*, and *synapsin* mRNA 2 h after medetomidine (0.3 mg/kg, i.p.) administration. Data are means ± S.E.M. *< 0.05, **< 0.01, ***< 0.001 (for statistical analyses and *n* numbers, see Table S2)

To test whether these molecular alterations are dependent on preceding cortical excitability, we subjected mice to an acute treatment with medetomidine, a hypnotic-sedative agent that directly facilitates slow EEG activity (Fig. 5a). However, TrkB^Y816^, p70S6k^T421/S424^, and GSK3β^S9^ phosphorylation levels were significantly increased also by this treatment (Fig. 5b) while it concomitantly brings unnoticeable acute effects on IEG expression and reduces gamma oscillations (Fig. 5c). This finding suggests that ongoing slow EEG activity, regardless of how it is regulated, positively correlates with molecular changes intimately connected with rapid antidepressant responses.

**Fig. 6:**
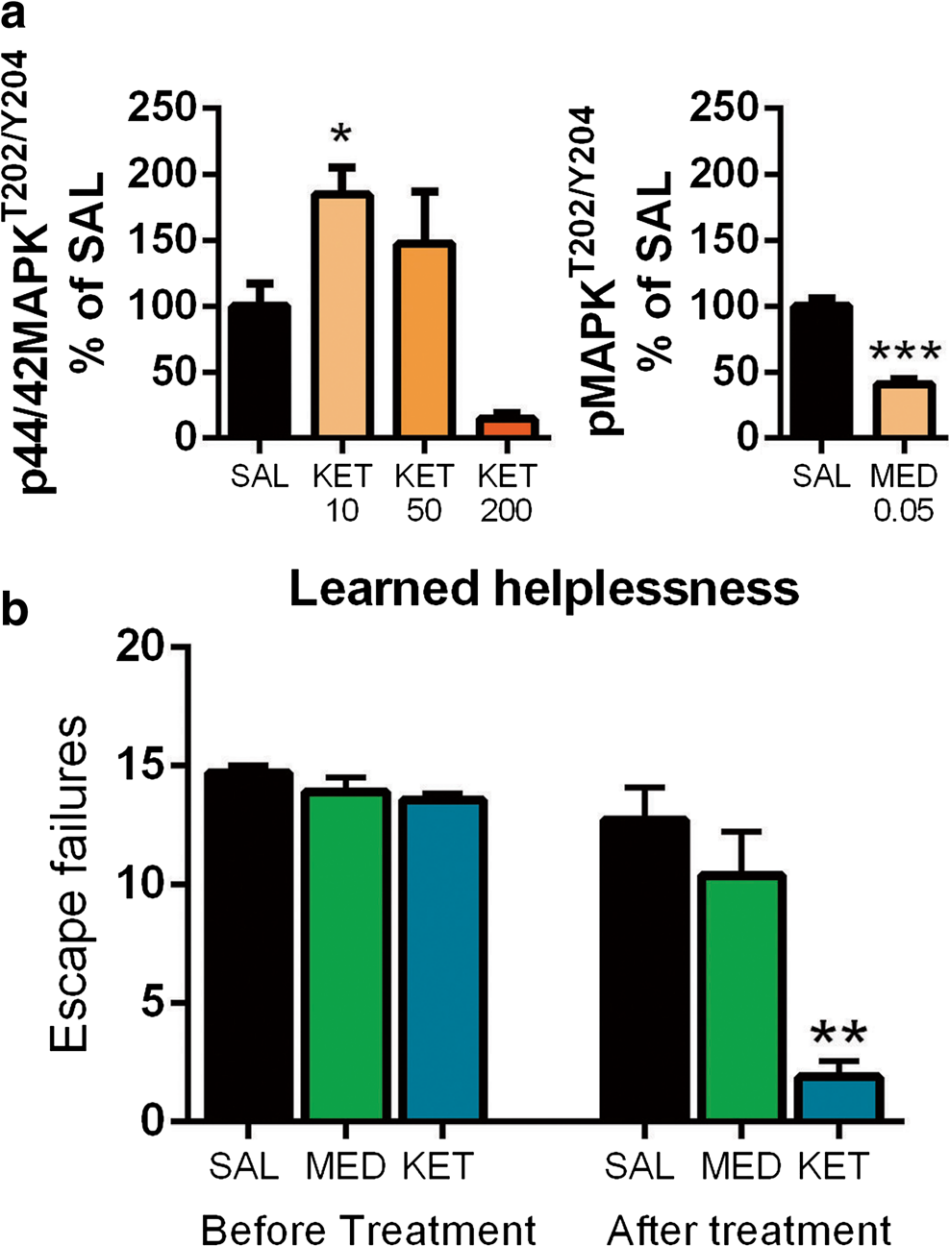
**a** Dose-dependent acute effects (30 min) of ketamine (KET) and effects of a low dose of medetomidine (MED; 0.05 mg/kg, i.p.) on phospho-MAPK^T202/Y204^. **b** Number of escape failures before and 24-h after low-dose ketamine (15 mg/kg, i.p.) or medetomidine (0.05 mg/kg, i.p.) in the learned helplessness paradigm. Data are means ± S.E.M. *< 0.05, ***< 0.001 (for statistical analyses and *n* numbers, see Table S2)

A single subanethetic dose of ketamine has been shown to produce rapid and long-lasting antidepressant-like behavioral changes [5, 39]. In addition to TrkB-mTor and GSK3β pathways, activation of MAPK signaling has been strongly implicated in the behavioral effects produced by antidepressants [44, 45]. While medetomidine readily regulates TrkB and GSK3β signaling, MAPK phosphorylation is strongly reduced by this treatment (Fig. 6a). It is thus tempting to speculate that the mechanisms of rapid antidepressant treatments are related to the combination of both excitation induced changes in gene expression and the subsequent homeostatic activation of key neurotrophic signaling pathways during postictal-like slow EEG oscillations. We tested this hypothesis with medetomidine in the learned helplessness paradigm, which has strong construct validity regarding depression. In this model, a rodent is exposed to inescapable mild foot shocks and subsequently tested for a deficit (helplessness) of acquired avoidance. A single subanesthetic dose of ketamine ameliorated the avoidance deficit within 24-h while medetomidine showed no such effect (Fig. 6b).

## Discussion

Despite great recent progress, the precise neurobiological basis governing rapid antidepressant effects remain obscure and debated [6, 17, 18, 46]. To get further insights into rapid antidepressant mechanisms, we investigated how N_2_O, another NMDA-R blocking dissociative anesthetic and a putative rapid-acting antidepressant [20], regulates EEG and the molecular level alterations previously implicated in rapid antidepressant mechanisms. The rapid pharmacokinetic properties of N_2_O allowed us to specifically and relatively precisely investigate these changes during the period of peak pharmacological effect and subsequently following NMDA-R blockade. Indeed, the antidepressant effects of subanesthetic ketamine become most evident few hours after infusion [3], at the time-point when the psychotomimetic effects of the drug have subsided.

Similarly to other clinically effective rapid-acting antidepressants, N_2_O increased biological markers related to increased cortical excitability. These changes, the upregulation of IEGs and the activating phosphorylation of p44/42-MAPK^T202/Y204^, appeared very rapidly and already during gas administration. In contrast, phosphorylation of TrkB^Y816^ and GSK3β^S9^ remained unaltered during N_2_O exposure and became regulated only gradually following drug discontinuation, during a brain state dominated by slow EEG activity and behavioral immobility. Importantly, and as previously shown, subanesthetic ketamine and flurothyl similarly evoked slow EEG oscillations that were generated after the peak of pharmacological effects had already passed. Moreover, TrkB and GSK3β signaling were robustly regulated during postictal slow EEG oscillations, a phenomenon previously associated with the efficacy and onset-of-action of ECT. While direct facilitation of slow EEG activity with medetomidine also produced robust phosphorylation of TrkB and GSK3β, no changes were present in the expression of IEGs and the phosphorylation of p44/42-MAPK was significantly decreased. Unlike ketamine, medetomidine failed to produce antidepressant-like behavioral responses in the learned helplessness paradigm. Taken together, these findings support a hypothesis that consecutive facilitation of cortical excitability and the regulation of TrkB and GSK3β signaling during the rebound slow EEG oscillations are essential for rapid antidepressant responses. Comparing the effects of rapid-acting antidepressants and medetomidine provides an excellent strategy to reveal the different neurobiological effects and phenomena set forth by direct vs. homeostatic facilitation of slow EEG oscillations and TrkB signaling.

This report proposes a novel link between a specific brain state, characterized by slow EEG oscillations and sedation, and the orchestrated and sequential regulation of multiple molecular targets implicated in rapid antidepressant responses. Furthermore, we demonstrate that this state and the accompanying regulation of molecular targets can be indirectly achieved by different excitatory interventions and speculate that this indirect homeostatically regulated state may be particularly important for rapid antidepressant effects. In a more general sense, this work urges shifting the attention towards understanding rapid antidepressant actions through alterations triggered within the brain as an adaptive consequence of a drug challenge. That said, interconnecting the present observations with the recently proposed effects of ketamine and other NMDA-R blockers on intrinsic homeostatic plasticity processes [9, 29, 30, 47–50], also evident as dynamic and circadian fluctuations in slow EEG oscillations [51–53], is instrumental in providing a more precise understanding of rapid antidepressant actions. Altogether, translating the emerging mechanistic evidence and hypotheses between cortical excitation and slow waves [54–57], such as the synaptic homeostasis hypothesis (SHY) of sleep [58, 59], to rapid antidepressant effects is essential in the future. The unique pharmacokinetic and pharmacological properties of N_2_O and related “fast-acting” medicines may become critical tools for these future efforts guiding the development of novel interventions against major depression.

## Electronic supplementary material

ESM 1 (DOCX 1526 kb)

## References

[1] Olesen J, Gustavsson A, Svensson M, Wittchen HU, Jönsson B (2012). The economic cost of brain disorders in Europe. Eur J Neurol.

[2] Jentsch Mike C, Van Buel Erin M, Bosker Fokko J, Gladkevich Anatoliy V, Klein Hans C, Oude Voshaar Richard C, Ruhé Henricus G, Eisel Uli LM, Schoevers Robert A (2015). Biomarker approaches in major depressive disorder evaluated in the context of current hypotheses. Biomarkers in Medicine.

[3] Aan Het Rot M, Zarate CA, Charney DS, Mathew SJ (2012). Ketamine for depression: where do we go from here?. Biol Psychiatry.

[4] Berman RM, Cappiello A, Anand A, Oren DA, Heninger GR, Charney DS, Krystal JH (2000). Antidepressant effects of ketamine in depressed patients. Soc Biol Psychiatry.

[5] Li N, Lee B, Liu R-J, Banasr M, Dwyer JM, Iwata M (2010). mTOR-dependent synapse formation underlies the rapid antidepressant effects of NMDA antagonists. Science.

[6] Duman RS, Aghajanian GK (2012). Synaptic dysfunction in depression: potential therapeutic targets. Science.

[7] Rantamäki T, Yalcin I (2016). Antidepressant drug action — From rapid changes on network function to network rewiring. Prog Neuropsychopharmacol Biol Psychiatry.

[8] Autry AE, Adachi M, Nosyreva E, Na ES, Los MF, Cheng P (2011). NMDA receptor blockade at rest triggers rapid behavioural antidepressant responses. Nature.

[9] Moghaddam B, Adams B, Verma A, Daly D (1997). Activation of glutamatergic neurotransmission by ketamine: a novel step in the pathway from NMDA receptor blockade to dopaminergic and cognitive disruptions associated with the prefrontal cortex. J Neurosci.

[10] Chowdhury G M I, Zhang J, Thomas M, Banasr M, Ma X, Pittman B, Bristow L, Schaeffer E, Duman R S, Rothman D L, Behar K L, Sanacora G (2016). Transiently increased glutamate cycling in rat PFC is associated with rapid onset of antidepressant-like effects. Molecular Psychiatry.

[11] Alt A, Nisenbaum ES, Bleakman D, Witkin JM (2006). A role for AMPA receptors in mood disorders. Biochem Pharmacol.

[12] Beurel E, Song L, Jope R (2011). Inhibition of glycogen synthase kinase-3 is necessary for the rapid antidepressant effect of ketamine in mice. Mol Psychiatry.

[13] Beurel E, Grieco SF, Amadei C, Downey K, Jope RS (2016). Ketamine-induced inhibition of glycogen synthase kinase-3 contributes to the augmentation of α-amino-3-hydroxy-5-methylisoxazole-4-propionic acid (AMPA) receptor signaling. Bipolar Disord.

[14] Zanos P, Gould TD (2018). Mechanisms of ketamine action as an antidepressant. Mol Psychiatry..

[15] Zanos P, Moaddel R, Morris PJ, Riggs LM, Highland JN, Georgiou P (2018). Ketamine and Ketamine Metabolite Pharmacology: Insights into Therapeutic Mechanisms. Pharmacol Rev.

[16] Zanos P, Moaddel R, Morris PJ, Georgiou P, Fischell J, Elmer GI (2016). NMDAR inhibition-independent antidepressant actions of ketamine metabolites. Nature.

[17] Hashimoto K, Shirayama Y (2017). What Are the Causes for Discrepancies of Antidepressant Actions of (2R,6R)-Hydroxynorketamine?. Biol Psychiatry.

[18] Collingridge GL, Lee Y, Bortolotto ZA, Kang H, Lodge D (2017). Antidepressant Actions of Ketamine Versus Hydroxynorketamine. Biol Psychiatry.

[19] Yamakura T, Harris RA (2000). Effects of gaseous anesthetics nitrous oxide and xenon on ligand-gated ion channels. Comparison with isoflurane and ethanol. Anesthesiology.

[20] Nagele P, Duma A, Kopec M, Gebara MA, Parsoei A, Walker M (2015). Nitrous Oxide for Treatment-Resistant Major Depression: A Proof-of-Concept Trial. Biol Psychiatry.

[21] Singh A, Kar SK (2017). How Electroconvulsive Therapy Works?: Understanding the Neurobiological Mechanisms. Clin Psychopharmacol Neurosci.

[22] Laurell B, Pern’s C (1970). Comparison of electric and flurothyl convulsive therapy: I. Seizure and post-seizure electroencephalographic pattern. Acta Psychiatr Scand.

[23] Fink M (2014). The seizure, not electricity, is essential in convulsive therapy: the flurothyl experience. J ECT.

[24] Wakamori M, Ikemoto Y, Akaike N (1991). Effects of two volatile anesthetics and a volatile convulsant on the excitatory and inhibitory amino acid responses in dissociated CNS neurons of the rat. J Neurophysiol.

[25] Sackeim HA, Luber B, Katzman GP, Moeller JR, Prudic J, Devanand DP, Nobler MS (1996). The effects of electroconvulsive therapy on quantitative electroencephalograms. Relationship to clinical outcome. Arch Gen Psychiatry.

[26] Feinberg I, Campbell IG (1995). Stimulation of NREM delta EEG by ketamine administration during waking: Demonstration of dose dependence. Neuropsychopharmacology.

[27] Feinberg I, Campbell IG (1993). Ketamine administration during waking increases delta EEG intensity in rat sleep. Neuropsychopharmacology.

[28] Campbell IG, Feinberg I (1996). NREM delta stimulation following MK-801 is a response of sleep systems. J Neurophysiol.

[29] Homayoun H, Moghaddam B (2007). NMDA receptor hypofunction produces opposite effects on prefrontal cortex interneurons and pyramidal neurons. J Neurosci.

[30] Widman AJ, McMahon LL (2018). Disinhibition of CA1 pyramidal cells by low-dose ketamine and other antagonists with rapid antidepressant efficacy. Proc Natl Acad Sci U S A.

[31] Yu X, Franks NP, Wisden W (2018). Sleep and Sedative States Induced by Targeting the Histamine and Noradrenergic Systems. Front Neural Circuits.

[32] Antila H, Ryazantseva M, Popova D, Sipilä P, Guirado R, Kohtala S (2017). Isoflurane produces antidepressant effects and induces TrkB signaling in rodents. Sci Rep.

[33] Kohtala S, Theilmann W, Suomi T, Wigren H-K, Porkka-Heiskanen T, Elo LL (2016). Brief Isoflurane Anesthesia Produces Prominent Phosphoproteomic Changes in the Adult Mouse Hippocampus. ACS Chem Neurosci.

[34] Dyrvig M, Christiansen SH, Woldbye DPD, Lichota J (2014). Temporal gene expression profile after acute electroconvulsive stimulation in the rat. Gene.

[35] Hansen HH, Rantamäki TPJ, Larsen MH, Woldbye DPD, Mikkelsen JD, Castrén EH (2007). Rapid activation of the extracellular signal-regulated kinase 1/2 (ERK1/2) signaling pathway by electroconvulsive shock in the rat prefrontal cortex is not associated with TrkB neurotrophin receptor activation. Cell Mol Neurobiol.

[36] Cirelli C, Pompeiano M, Tononi G (1995). Sleep deprivation and c-fos expression in the rat brain. J Sleep Res.

[37] Hiyoshi T, Kambe D, Karasawa J, Chaki S (2014). Differential effects of NMDA receptor antagonists at lower and higher doses on basal gamma band oscillation power in rat cortical electroencephalograms. Neuropharmacology.

[38] Maxwell CR, Ehrlichman RS, Liang Y, Trief D, Kanes SJ, Karp J, Siegel SJ (2006). Ketamine produces lasting disruptions in encoding of sensory stimuli. J Pharmacol Exp Ther.

[39] Sun H-L, Zhou Z-Q, Zhang G-F, Yang C, Wang X-M, Shen J-C (2016). Role of hippocampal p11 in the sustained antidepressant effect of ketamine in the chronic unpredictable mild stress model. Transl Psychiatry.

[40] Saarelainen T, Hendolin P, Lucas G, Koponen E, Sairanen M, MacDonald E (2003). Activation of the TrkB neurotrophin receptor is induced by antidepressant drugs and is required for antidepressant-induced behavioral effects. J Neurosci.

[41] Rantamäki Tomi, Hendolin Panu, Kankaanpää Aino, Mijatovic Jelena, Piepponen Petteri, Domenici Enrico, Chao Moses V, Männistö Pekka T, Castrén Eero (2007). Pharmacologically Diverse Antidepressants Rapidly Activate Brain-Derived Neurotrophic Factor Receptor TrkB and Induce Phospholipase-Cγ Signaling Pathways in Mouse Brain. Neuropsychopharmacology.

[42] Huang EJ, Reichardt LF (2001). Neurotrophins: roles in neuronal development and function. Annu Rev Neurosci.

[43] Di Lieto Antonio, Rantamäki Tomi, Vesa Liisa, Yanpallewar Sudhirkumar, Antila Hanna, Lindholm Jesse, Rios Maribel, Tessarollo Lino, Castrén Eero (2012). The Responsiveness of TrkB to BDNF and Antidepressant Drugs Is Differentially Regulated during Mouse Development. PLoS ONE.

[44] Réus GZ, Vieira FG, Abelaira HM, Michels M, Tomaz DB, dos Santos MAB (2014). MAPK signaling correlates with the antidepressant effects of ketamine. J Psychiatr Res.

[45] Duman CH, Schlesinger L, Kodama M, Russell DS, Duman RS (2007). A role for MAP kinase signaling in behavioral models of depression and antidepressant treatment. Biol Psychiatry.

[46] Zanos P, Thompson SM, Duman RS, Zarate CA, Gould TD (2018). Convergent Mechanisms Underlying Rapid Antidepressant Action. CNS Drugs.

[47] Nosyreva E, Szabla K, Autry AE, Ryazanov AG, Monteggia LM, Kavalali ET (2013). Acute Suppression of Spontaneous Neurotransmission Drives Synaptic Potentiation. J Neurosci.

[48] Miller OH, Yang L, Wang C-C, Hargroder EA, Zhang Y, Delpire E, Hall BJ (2014). GluN2B-containing NMDA receptors regulate depression-like behavior and are critical for the rapid antidepressant actions of ketamine. eLife.

[49] Sutton MA, Ito HT, Cressy P, Kempf C, Woo JC, Schuman EM (2006). Miniature neurotransmission stabilizes synaptic function via tonic suppression of local dendritic protein synthesis. Cell.

[50] Workman ER, Niere F, Raab-Graham KF (2017). Engaging homeostatic plasticity to treat depression. Mol Psychiatry.

[51] Duncan WC, Zarate CA (2013). Ketamine, sleep, and depression: current status and new questions. Curr Psychiatry Rep.

[52] Duncan WC, Selter J, Brutsche N, Sarasso S, Zarate CA (2013). Baseline delta sleep ratio predicts acute ketamine mood response in major depressive disorder. J Affect Disord.

[53] Duncan WC, Sarasso S, Ferrarelli F, Selter J, Riedner BA, Hejazi NS (2013). Concomitant BDNF and sleep slow wave changes indicate ketamine-induced plasticity in major depressive disorder. Int J Neuropsychopharmacol.

[54] Huber R, Ghilardi MF, Massimini M, Ferrarelli F, Riedner BA, Peterson MJ, Tononi G (2006). Arm immobilization causes cortical plastic changes and locally decreases sleep slow wave activity. Nat Neurosci.

[55] Huber R, Esser SK, Ferrarelli F, Massimini M, Peterson MJ, Tononi G (2007). TMS-induced cortical potentiation during wakefulness locally increases slow wave activity during sleep. PloS One.

[56] Huber R, Deboer T, Tobler I (2000). Topography of EEG dynamics after sleep deprivation in mice. J Neurophysiol.

[57] Kattler H, Dijk DJ, Borbély AA (1994). Effect of unilateral somatosensory stimulation prior to sleep on the sleep EEG in humans. J Sleep Res.

[58] Tononi G, Cirelli C (2003). Sleep and synaptic homeostasis: a hypothesis. Brain Res Bull.

[59] Tononi G, Cirelli C (2014). Sleep and the Price of Plasticity: From Synaptic and Cellular Homeostasis to Memory Consolidation and Integration. Neuron.

