# Supplementary material for: Cortical Excitability and Activation of TrkB Signaling During Rebound Slow Oscillations Are Critical for Rapid Antidepressant Responses
Source: Mol Neurobiol. 2018 Oct 4;56(6):4163–74. doi: 10.1007/s12035-018-1364-6 (PMC6505519; doi:10.1007/s12035-018-1364-6)
Supplement: Supplementary file 1 — (DOCX 1526 kb) [file 12035_2018_1364_MOESM1_ESM.docx]

Supplementary Material

**Cortical excitability and activation of TrkB signaling during rebound slow oscillations are critical for rapid antidepressant responses**

Samuel Kohtala^1,2^, Wiebke Theilmann^1#^, Marko Rosenholm^1,2#^, Leena Penna^1^, Gulsum Karabulut^3,4^, Salla Uusitalo^1,2^, Kaija Järventausta^5^, Arvi Yli-Hankala^6,7^, Ipek Yalcin^3^, Nobuaki Matsui^8^, Henna-Kaisa Wigren^9^ and Tomi Rantamäki^1,2^*

^1^Laboratory of Neurotherapeutics, Faculty of Biological and Environmental Sciences, P.O.Box 65 (Viikinkaari 1), University of Helsinki, Finland

^2^Faculty of Pharmacy, Division of Pharmacology and Pharmacotherapy, University of Helsinki, Finland

^3^Institut des Neurosciences Cellulaires et Intégratives, Centre National de la Recherche Scientifique and Université de Strasbourg, FR-67000 Strasbourg Cedex, France.

^4^Department of Anesthesiology and Reanimation, Gazi University, Turkey

^5^Department of Psychiatry, Tampere University Hospital, Finland

^6^Faculty of Medicine and Life Sciences, Department of Anesthesiology, University of Tampere

^7^Department of Anaesthesia, Tampere University Hospital, Finland

^8^Faculty of Pharmaceutical Sciences, Tokushima Bunri University, Tokushima, Japan

^9^Faculty of Medicine, Medicum/Physiology, University of Helsinki, FI-00014 Helsinki, Finland

**List of Supplementary Materials**

- Figures S1–S2
- Tables S1-S2
- References for supplementary materials


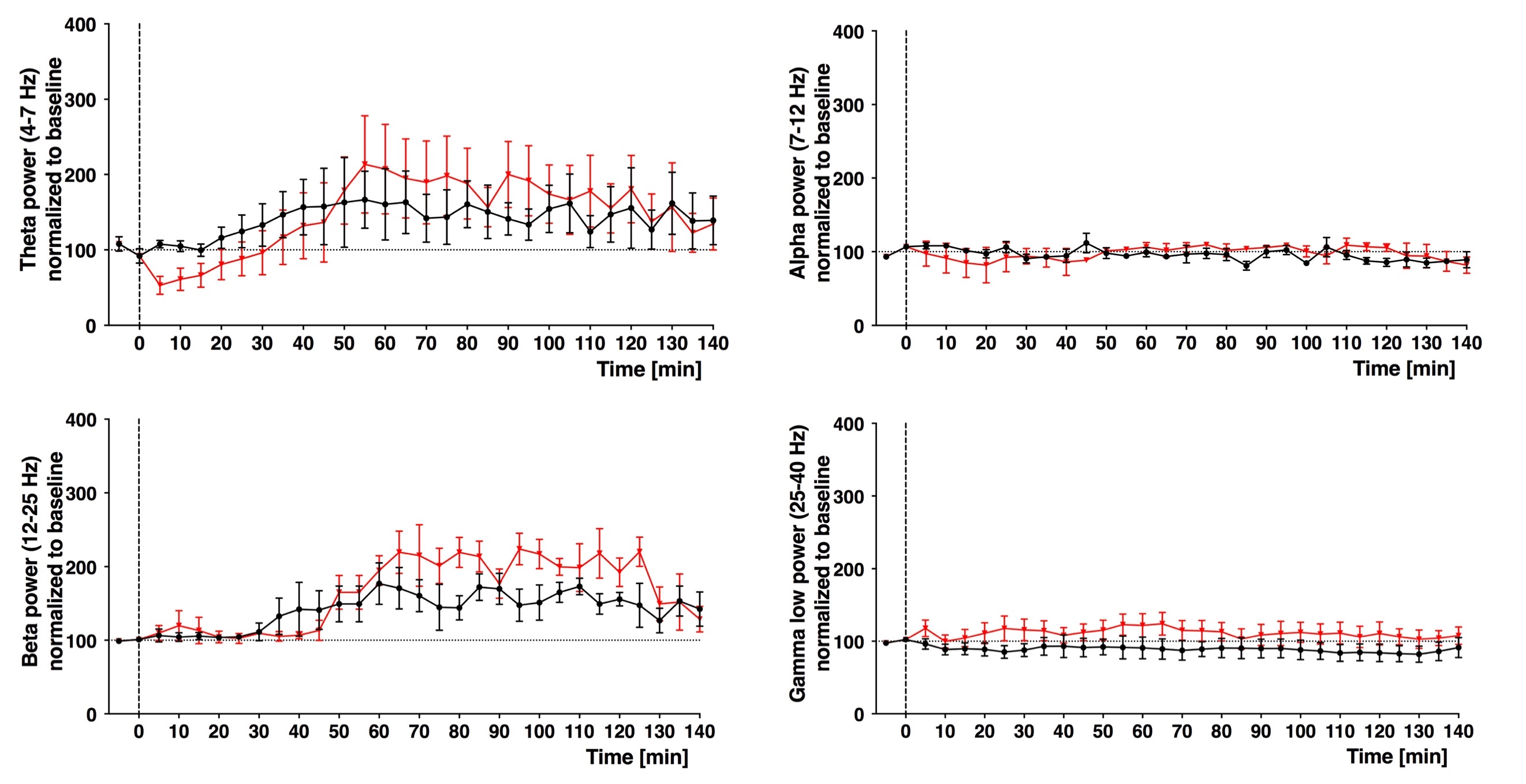


**Figure S1.** Theta, alpha, beta and low gamma oscillations after an acute injection of saline (black) or subanesthetic ketamine (red; 10 mg/kg, i.p.). Data analyzed in 5 min bins. N=4/group.


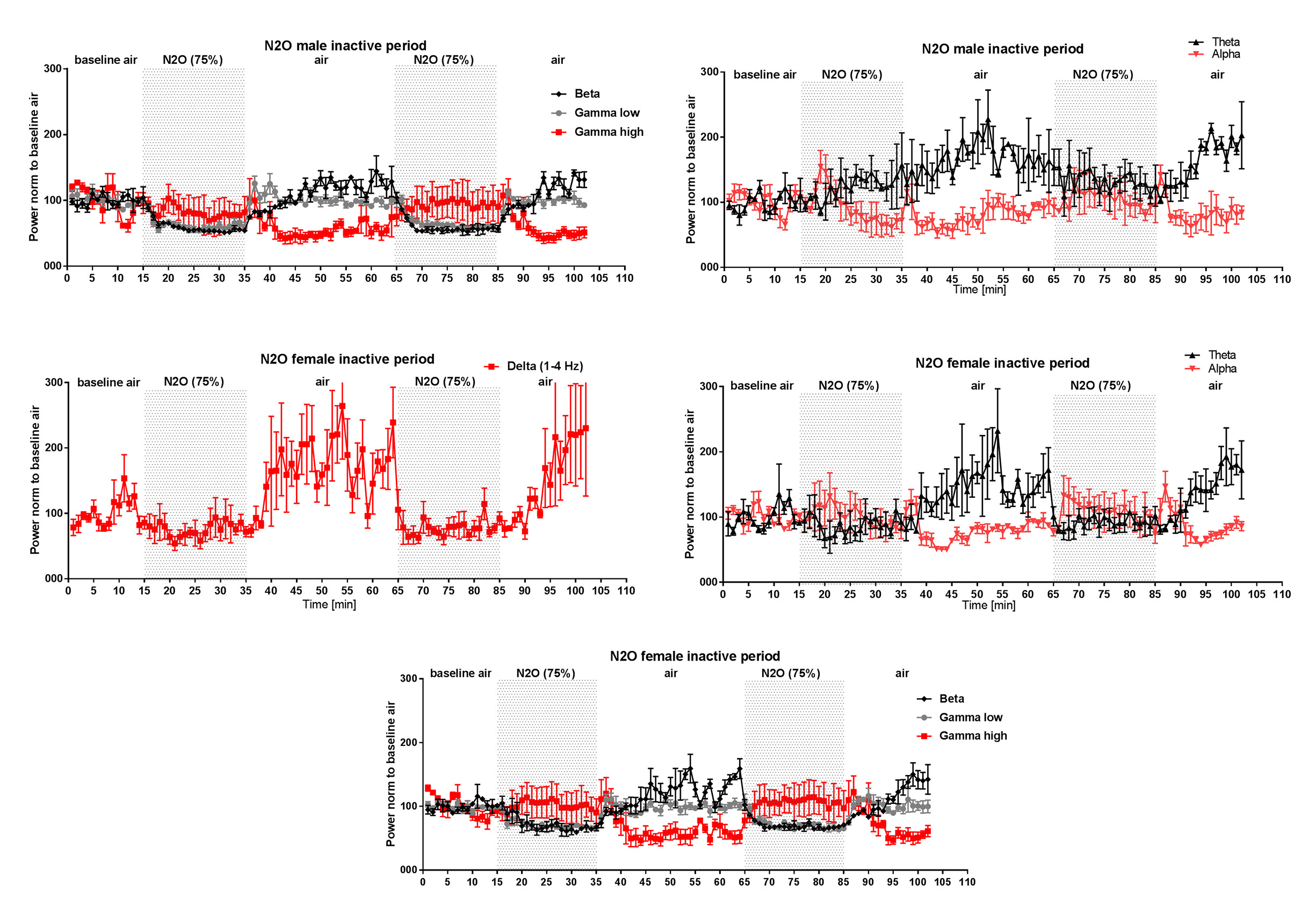


**Figure S2.** (**A**) Normalized power of beta, gamma, theta and alpha oscillations in male mice before, during (grey background) and after N_2_O (75%). (**B**) Normalized power of major EEG oscillations in female mice before, during (grey background) and after N_2_O treatment (75%). Data analyzed in 1 min bins. N=3/group. Data are means ± S.E.M.

| ***Gene*** | ***Forward primer*** | ***Reverse primer*** | ***Reference*** |
| --- | --- | --- | --- |
| ***Arc*** | AAGTGCCGAGCTGAGATGC | CGACCTGTGCAACCCTTTC | Primer bank ID 9055166a1 |
| ***ß-actin*** | GGCTGTATTCCCCTCCATCG | CCAGTTGGTAACAATGCCATGT | Primer bank ID 6671509a1 |
| ***Bdnf (exon IV)*** | ACCGAAGTATGAAATAACCATAGTAAG | TGTTTACTTTGACAAGTAGTGACTGAA | Ref. 1 |
| ***Bdnf (total)*** | GAAGGCTGCAGGGGCATAGACAAA | TACACAGGAAGTGTCTATCCTTATG | Ref. 1 |
| ***cFos*** | CGGGTTTCAACGCCGACTA | TTGGCACTAGAGACGGACAGA | Primer bank ID 6753894a1 |
| ***Egr1*** | GCCAAGGCCGTAGACAAAATC | CCACTCCGTTCATCTGGTCA | Primer bank ID 14318592a1 |
| ***Gapdh*** | GGTGAAGGTCGGTGTGAACGG | CATGTAGTTGAGGTCAATGAAGGG | Ref. 2 |
| ***Homer1a*** | GGCAAACACTGTTTATGGACTGG | GTAATTCAGTCAACTTGAGCAACC | Ref. 3 |
| ***Mkp1*** | CTGCTTTGATCAACGTCTCG | AAGCTGAAGTTGGGGGAGAT | Ref. 4 |
| ***Synapsin*** | ACACCGACTGGGCAAAATA | GTCACAGAAGTTGTAGACAGAATG |  |
| ***Zif268 (egr-1)*** | TCCTCTCCATCACATGCCTG | CACTCTGACACATGCTCCAG | Ref. 5 |

**Table S1.** Primers used for quantitative RT-PCR.

**Table S2.** Statistical tests and n-numbers for main figures.

**References**

1 Karpova NN, Rantamäki T, Di Lieto A, Lindemann L, Hoener MC, Castrén E. Darkness reduces BDNF expression in the visual cortex and induces repressive chromatin remodeling at the BDNF gene in both hippocampus and visual cortex. *Cell Mol Neurobiol* 2010; **30**: 1117–1123.

2 Rantamäki T, Kemppainen S, Autio H, Stavén S, Koivisto H, Kojima M *et al.* The Impact of Bdnf Gene Deficiency to the Memory Impairment and Brain Pathology of APPswe/PS1dE9 Mouse Model of Alzheimer’s Disease. *PLoS ONE* 2013; **8**: e68722.

3 Luo P, Chen T, Zhao Y, Zhang L, Yang Y, Liu W *et al.* Postsynaptic scaffold protein Homer 1a protects against traumatic brain injury via regulating group I metabotropic glutamate receptors. *Cell Death Dis* 2014; **5**: e1174.

4 Taylor DM, Moser R, Régulier E, Breuillaud L, Dixon M, Beesen AA *et al.* MAP kinase phosphatase 1 (MKP-1/DUSP1) is neuroprotective in Huntington’s disease via additive effects of JNK and p38 inhibition. *J Neurosci Off J Soc Neurosci* 2013; **33**: 2313–2325.

5 Hendrickx A, Pierrot N, Tasiaux B, Schakman O, Brion J-P, Kienlen-Campard P *et al.* Epigenetic induction of EGR-1 expression by the amyloid precursor protein during exposure to novelty. *PloS One* 2013; **8**: e74305.
